# Does COVID-19 threat increase xenophobia? The roles of protection efficacy and support seeking

**DOI:** 10.1186/s12889-022-12912-8

**Published:** 2022-03-11

**Authors:** Zhuang She, Ningning Zhou, Dan Li, Shengtao Ren, Weidong Ji, Juzhe Xi

**Affiliations:** 1 Shanghai Key Laboratory of Mental Health and Psychological Crisis Intervention, Affiliated Mental Health Center (ECNU), Juzhe Xi’s Master Workroom of Shanghai School Mental Health Service, School of Psychology and Cognitive Science, East China Normal University, Shanghai, 200062 China; 2 Shanghai Changning Mental Health Center, Shanghai, 200335 China

**Keywords:** COVID-19, Disease threats, Protection efficacy, Support seeking, Xenophobia in China

## Abstract

**Background:**

In response to the COVID-19 pandemic, people in many countries have shown xenophobia toward China, where the pandemic began. Within China, xenophobia has also been observed toward the people of Wuhan, the city where the first cases were identified. The relationship between disease threat and xenophobia is well established, but the reasons for this relationship are unclear. This study investigated the mediation role of perceived protection efficacy and moderation role of support seeking in the relationship between perceived COVID-19 risk and xenophobia within China.

**Methods:**

An online survey was administered to a nationally representative sample (*N* = 1103; 51.7% women; ages 18 to 88) of Chinese adults during the early stage of the COVID-19 pandemic. Participants completed questionnaires about their perceived COVID-19 risk, perceived protection efficacy in reducing risk, support seeking, and xenophobic attitudes toward people of the Wuhan area.

**Results:**

Regression based analyses showed that the perceived COVID-19 risk positively predicted xenophobia. Low perceived protection efficacy partly mediated the relationship between perceived COVID-19 risk and xenophobic attitudes, and this indirect effect was moderated by support seeking. Specifically, the indirect effect was weaker among individuals who sought more social support.

**Conclusions:**

Under disease threat, xenophobia can appear within a country that otherwise seems culturally homogeneous. This study extends the extant research by identifying a possible psychological mechanism by which individuals’ perception of disease threat elicits xenophobia, and by addressing the question of why this response is stronger among some people than others. Increasing the public’s perceived efficacy in protecting themselves from infection, and encouraging support seeking, could reduce xenophobic attitudes.

## Background

Many people have shown xenophobia toward China in response to the COVID-19 pandemic [1]. The concept of xenophobia describes prejudice towards those who are perceived as a threat, such as foreigners and outgroups [2]. Xenophobia increases during periods of disease threat [2, 3] because outsiders are believed to be carriers of germs and infections. To avoid potential infections, people tend to express negative attitudes towards outgroup members and to avoid them [2]. However, xenophobia is widely regarded as an undesirable response to disease threat, as it can have negative effects not just on targets but also on the political destabilization within in a society and tensions among nations [4, 5]. Meanwhile, xenophobia also creates a greater psychological burden on those who are most at-risk for illness [6], thus exacerbating the consequences of infectious diseases.

However, xenophobia has also been expressed within China toward the people of Wuhan, the city that was the epicenter of the pandemic [7]. To our knowledge, no studies have systematically investigated xenophobic attitudes under disease threat within a country. The current COVID-19 crisis provided us a unique opportunity to explore xenophobia within China as the Chinese expressed it toward the people of Wuhan. Given that xenophobia under the COVID-19 crisis would bring about additional burdens on individuals, societies and nations, more studies are needed to address this issue. For example, research on the underlying mediating mechanism (i.e., how COVID-19 risk influences xenophobia) and moderating factors (i.e., the conditions under which the mediating process is most potent) would inform the development of targeted early interventions to reduce the effect of perceived COVID-19 threat on xenophobia. To this end, we drew from existing literature to propose and test a model that would provide this kind of information. Specifically, we investigated whether the relationship between COVID-19 risk and xenophobia is due in part to low perceived protection efficacy, and whether this indirect relationship is weakened by support seeking.

### The relationship between disease threat and xenophobia

Numerous studies have examined the relationship between disease threat and xenophobia. These include early laboratory studies [3], research on chronic disease threat [8, 9], as well as research in the context of realistic disease threats (e.g., Ebola, avian influenza) [9, 10]. The findings of these studies consistently suggested that disease threats were linked to an increased xenophobic response. In other words, people’s perceived disease threat positively predicts their xenophobic response toward a certain group (e.g., immigrants) [3, 10]. However, the available studies have been conducted on xenophobia toward immigrants in Western cultures, such as U.S. residents’ xenophobia toward West Africans and undocumented immigrants during the Ebola outbreak [10] and Swiss residents’ xenophobia toward foreigners in Switzerland during the avian influenza crisis [8].

### The mediating role of protection efficacy

There is little research on factors that might explain the association between perceived disease threat and xenophobic attitudes. One study found that ideological and normative beliefs (e.g., social dominance orientation, belief in a dangerous world) mediated the relationship between the perceived threat of chronic disease (germ aversion) and exclusionary immigration attitudes [9]. However, these mediation relationships were not found during an avian influenza pandemic [9]. Another study found that perceived protection efficacy mediated the relationship between perceived Ebola threat and xenophobia [10].

In the current study we tested protection efficacy as a mediator of the association between perceived disease risk and xenophobia during the COVID-19 pandemic. The Protection Motivation Theory (PMT) [11] offers a framework for understanding how disease threat is transformed into xenophobic attitudes. According to the PMT, an individual’s perception of a health threat is determined by various cognitive appraisal processes, including their perceived vulnerability to the risk and their ability to cope with it. Specifically, people will feel threatened psychologically if they believe they are vulnerable to a threat but lack the ability to protect themselves from it [10, 11]. Additionally, the research on learned helplessness in mental health also suggested that uncontrollable environmental stressors or aversive events were associated with low self-efficacy [12]. This evidence supported the relationship between the COVID-19 risk (an inescapable stressful event) and lower protection efficacy perceived by individuals. However, it is favorable for people to avert the threats if they adopt the appropriate attitudes and act upon them [13]. Following the PMT, under the COVID-19 threat, people may first assess their vulnerability to COVID-19 and then evaluate the degree of how much they and members of their group could protect themselves from the COVID-19. If they perceived that they and their communities could not defend themselves from COVID-19 threat (low protection efficacy), one self-protection strategy was to establish response patterns that might prevent the infection risk; for example, keeping the people from areas most affected by an epidemic (e.g., people from the Wuhan in this study) at a distance and showing xenophobia attitude toward them under the COVID-19 threat.

Based on PMT, a previous study investigated the relationships among perceived Ebola risk, perceived protection efficacy and xenophobia. The results showed that higher Ebola risk was associated with lower perceived protection efficacy, which was further associated with increased xenophobic attitudes [10]. We hoped to confirm this finding and extend it to the context of the COVID-19 crisis (early stage in this study). According to the PMT and existing research, we hypothesized that perceived protection efficacy might mediate the relationship between perceived COVID–19 risk and a xenophobic response.

### The moderating role of support seeking

There is little research on factors that might make some people more or less likely to show xenophobia in response to perceived disease threat. This type of research would provide information about protective factors, or buffers, that reduce the risk of xenophobia even in the face of disease threat. The only study to date on this issue found that individuals with strong collectivistic attitudes showed less of a xenophobic response under the Ebola crisis [10]. Whether there are other protective factors remains unknown. In addition, factors that may be protective against general xenophobia may not be applicable in the context of infectious disease, with fear of intergroup contact [14]. More studies are needed to address this issue.

In the current study we tested support seeking as a moderator of the indirect relationship between perceived disease threat and xenophobic attitudes. That is, social support seeking, as a coping strategy, should protect against the influence of disease threat in lowering perceived protection efficacy, and this should further reduce the xenophobic response. Social support seeking is conceptualized as one’s active efforts to gain support from others [15]. In the existing literature, this concept is consistently regarded as an adaptive and positive strategy to cope with stressful events [16–18]. Recent studies suggested that a support seeking strategy was widely used during the SARS [19] and COVID-19 outbreaks [20]. In contrast to someone who uses an avoidance coping strategy, a person who uses support seeking attempts to actively change a stressful situation [15]. To this end, individuals may seek support of multiple types (e.g., emotional and instrumental support) and from multiple sources (e.g., family, friends or other helpers) [15, 21].

Although support seeking has demonstrated a positive effect in the literature in terms of mental health, few studies have examined the buffering effect of support seeking in the context of disease threat. Research has shown that having more support is related to greater well-being for people under stress, as support resources can service as stress buffers and protect individuals from the potential negative influence of stressful events [22]. Given that disease threat can be considered a significant stressor [19], we expected that the strength of the association between perceived COVID-19 risk and low protection efficacy would be reduced by support seeking, thus affecting the overall mediation process. Specifically, under the same perceived COVID-19 risk, people seeking more social support would perceive higher protection efficacy. In other words, support seeking may promote people’s perceived efficacy to protect themselves from COVID-19 threat. This idea is consistent with research showing that more support could be translated into greater self-efficacy, especially at moderate to high levels of stress [23].

Although we expected that support seeking would buffer the association between disease threat and protection efficacy (path a), we did not expect that it would buffer the direct link between perceived COVID-19 threat and xenophobia (path c), as well as the second part of the indirect effect (path b). That is because, even although it is well established that intergroup interactions decrease prejudices [24]; and support seeking may occur through interactions with others; nevertheless, support seeking interactions tend to occur among known in-group members (e.g., family and friends) [15] rather than with out-group members (e.g., people from Wuhan). Thus, in theory, support seeking would not be expected to lessen xenophobia directly. For the same reason, we also did not expect that support would moderate the relationship between perceived protection efficacy and xenophobia (path b). In the present study, we hypothesized only that support seeking would moderate the first path of the mediating effect.

### The present study

The present study was conducted in China during the first few months of the COVID-19 crisis. The disease was first reported in Wuhan, China, and soon spread throughout China due to the mass movement during the country’s Spring Festival travel rush. It was reported that more than 5 million people had already left Wuhan before the city was locked down [25]. Thus, people recognized that contact with people from Wuhan was the main cause of contracting COVID-19 [26]. Due to worldwide media attention, people from Wuhan aroused great fear in the public all over China and even the world. Given that xenophobia in response to disease threat is based on fear of unknown pathogens from outgroups [3], and the Chinese public reported increased negative attitudes toward Wuhan people during this time [7], we considered the Wuhan people as a special “outgroup” and defined xenophobia as the Chinese public’s prejudiced attitude toward the Wuhan people.

**Fig. 1:**
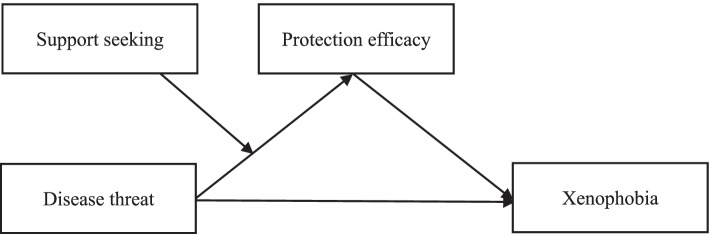
Moderated mediation model

In sum, this study examined whether the relationship between disease threat and individuals’ xenophobic attitudes was mediated by protection efficacy, and assessed whether support seeking played a moderation role in the first part of the mediating process (the association between perceived disease threat and protection efficacy). The proposed integrated model is shown in Fig. 1. The following hypotheses were tested:

- Hypothesis 1. Perceived COVID-19 risk will positively predict xenophobia.
- Hypothesis 2. Low protection efficacy will mediate the relationship between perceived COVID-19 risk and xenophobia.
- Hypothesis 3. Support seeking will moderate the first link of the indirect effect of perceived COVID-19 risk on xenophobia, such that the indirect effect will be weaker for individuals who report more support seeking.

## Methods

### Participants and procedure

The commercial online survey research firm Wenjuanxing (https://www.wjx.cn/) was employed to construct a nationally representative sample between February 25 and March 2, 2020, approximately 1 month after the massive outbreak of COVID-19 in China. During this early phase of the pandemic the severity and infection risk of COVID-19 in China was much higher than in other countries. Chinese citizens who had reached 18 years of age were contacted to participate in the survey. We obtained 1133 response sets in the given period. This number excluded data from participants who dropped out of the study after completing only part of the survey. Given that our research interest was the Chinese public’s attitude toward people from the city of Wuhan, 30 participants from Wuhan were excluded from the final analysis. Therefore, the final sample included 1103 participants (533 men, 570 women). The average age was 31.29 years (SD = 8.77), ranging from 18 to 88 years. Almost 9.2% of participants had a master’s or doctorate degree, 64.8% had a college degree, 14.9% had an associate degree, and 11.1% had a high school degree or below.

Using the online interface, participants were firstly informed about the research aim, the general topics of the questionnaires, and the estimated completion time. They were also informed that their responses were anonymous and would only be used for research purposes. If participants gave consent to participate, they were then directed to the online research questionnaires. To eliminate the problem of missing data, participants had to give a response to each question before moving to the next. Participants were informed that if they felt any discomfort while answering, they could choose to withdraw from the study at any time. All participants who completed the questionnaires received reward points that could be converted into cash (about USD 0.8 for this survey). This study was approved by the East China Normal University research ethics committee (HR151–2020).

### Measures

The questionnaires were identified in English language publications and were adapted for purposes of the current research (for example, references to Ebola were changed to refer to COVID-19, for more details see below). All items were translated from English into Chinese following a cross-cultural translation protocol [27]. Both exploratory factor analysis (EFA, based on the first half of the data) and confirmatory factor analysis (CFA, based on the second half of the data) were used to examine the construct validity of the measures.

### Perceived vulnerability to COVID-19 risk

A nine-item scale that was developed to measure the perceived risk of Ebola [10] was adapted to assess the perceived risk of COVID-19 by changing “Ebola” to “COVID-19”. The measure consists of three 3-item subscales measuring (a) perceptions of personal risk (e.g., “I feel vulnerable to COVID-19 infection”), (b) perceptions of the local community’s risk (e.g., “I feel that people in my local community are vulnerable to COVID-19 infection”), and (c) perceptions of risk to the city (e.g., “I feel that my city is vulnerable to an outbreak of COVID-19”). Participants rated the items using a 5-point scale from 1 (strongly disagree/never) to 5 (strongly agree/all the time). EFA indicated that the nine items of the perceived risk of COVID-19 scale loaded on two factors: concern about infection (6 items) and perceived vulnerability (3 items). CFA showed a good fit to the data: CFI = 0.951, TLI = 0.929, RMSEA = 0.086. The nine items were averaged to create a composite score, and higher scores indicated the perception of higher disease risk due to COVID-19. The internal consistency of the scale in the current sample was satisfactory (α = 0.84).

### Protection efficacy

A six-item scale that was developed to assess perceived protection efficacy by individuals under the Ebola threat [10] was adapted to assess the same perception under the COVID-19 threat. Two items measure the perception of personal protection efficacy (e.g., “I feel confident that I can protect myself from COVID-19”), two items measure the perception of the community’s protection efficacy (e.g., “I feel confident that my local community can protect itself from COVID-19”), and two items measure the perception of the city’s protection efficacy (e.g., “I feel confident that my city can protect itself from COVID-19”). All items were scored on a 7–point Likert scale from 1 (strongly disagree) to 7 (strongly agree). EFA indicated that the six items of the protection efficacy scale loaded on two factors: perceived positive efficacy (3 items) and perceived negative efficacy (3 items). The index of CFA showed a good fit to the data: CFI = 0.964, TLI = 0.932, RMSEA = 0.079. The composite score was calculated by reverse-coding three items and averaging the scores for all items, with higher scores indicating perceived greater protection efficacy. The internal consistency of the scale in the current sample was slightly lower than in past research [10] but still good (α = 0.70).

### Support seeking

Support seeking was adapted from the six-item seeking social support subscale of the Coping Questionnaire [15]. Rather than reporting their general efforts at seeking social support, the participants reported their efforts at seeking social support specifically related to the COVID-19 epidemic. Participants were asked to rate their efforts to seek instrumental support (e.g., “Talking to someone who could do something concrete about COVID-19”) and emotional support (e.g., “Talking to someone about how I was feeling about COVID-19”) during the COVID–19 epidemic, using a 4-point scale from 1 (not at all) to 4 (a great deal). EFA indicated that the six items of the support seeking scale loaded on two factors corresponding to instrumental (3 items) and emotional support (3 items). The index of CFA showed a marginally acceptable fit to the data: CFI = 0.943, TLI = 0.893, RMSEA = 0.101. The six items were averaged to create a composite score, and higher scores indicated more support seeking. The internal consistency of the scale in the current sample was α = 0.72, which is comparable to the past research [15].

### Xenophobia

The measure of xenophobia was adapted from the 10-item Prejudiced Attitude Scale [28]. The Chinese public’s prejudice toward people from Wuhan was used to operationalize xenophobia and was assessed by asking participants to rate their feelings toward this group of people (Instruction: “When there are people from Wuhan around you, or if you imagine it, what is your feeling towards them?”). The ratings were made with reference to (a) 5 negative attitudes: hostility, disliking, hatred, rejection, fear; (b) 5 positive attitudes: acceptance, affection, approval, sympathy, and warmth). Ratings were made using an 8-point scale from 0 (do not feel this emotion at all) to 7 (feel this emotion strongly). EFA indicated that the ten items of the xenophobia measure loaded on two factors corresponding to negative (5 items) and positive attitudes (5 items). CFA showed an acceptable fit: CFI = 0.928, TLI = 0.904, RMSEA = 0.095. The composite score was calculated by reverse-coding the five positive emotion items and averaging the scores for all items. Higher scores indicated greater xenophobia toward the people of Wuhan. The internal consistency in the current sample was α = 0.84.

### Statistical analysis

We first conducted correlation analyses and calculated descriptive statistics with SPSS 21.0. Then, we used the SPSS macro PROCESS [29], which was developed to test complex models including both mediator and moderator variables, to test our proposed model. Model 4 was used to test the predicted indirect effect (mediating effect) from perceived COVID-19 threat to xenophobia through protection efficacy. We also used Model 7 to test whether support seeking moderated the indirect effect of perceived COVID-19 threat on xenophobia. Bootstrapping was applied with 5000 samples, and bias–corrected 95% confidence intervals (CIs) were used to examine the mediation effect and the moderated mediation effect. Given the evidence that women and younger adults exhibit more xenophobia [30], we controlled for gender and age as potential confounding variables in the analyses.

## Results

### Preliminary analyses

Means, standard deviations, and correlations among the variables are presented in Table 1. Perceived COVID-19 threat was positively correlated with support seeking and xenophobia but negatively correlated with perceived protection efficacy; perceived protection efficacy was negatively correlated with xenophobia; and support seeking was negatively correlated with xenophobia. These descriptive results provided preliminary support for the hypotheses.

**Table 1:** Descriptive statistics and correlation for study variables (*N* = 1103)

Variables	*M*	*SD*	3	4	5	6
1. Gender	–	–				
2. Age	31.29	8.77				
3. Disease threat	3.08	0.69	–			
4. Protection efficacy	4.83	0.98	−0.41^***^	–		
5. Support seeking	2.69	0.57	0.24^***^	0.05	–	
6. Xenophobia	4.14	1.24	0.16^***^	−0.12^***^	−0.12^***^	–

^***^p < 0.001; Disease threat: perceived COVID-19 threat (the same below)

### Test of mediation model

After controlling for gender and age, perceived COVID-19 threat positively predicted xenophobia (*β* = 0.24, *p* < 0.001), supporting H1. Perceived COVID–19 threat negatively predicted perceived protection efficacy (*β* = − 0.62, *p* < 0 .001), and perceived protection efficacy negatively predicted xenophobia (*β* = − 0.09, *p* < 0.05). A bias–corrected bootstrap with 5000 samples estimated two effects: direct effect (effect of perceived COVID-19 threat on xenophobia), and mediation effect (effect of protection efficacy in the relationship between perceived COVID-19 threat and xenophobia). There was a significant mediation effect (B = 0.05, 95% CI [0.01, 0.10]) and direct effect (B = 0.24, 95% CI [0.12, 0.36]). Therefore, protection efficacy partially mediated the relation between perceived COVID-19 threat and xenophobia. These results provided support for H2.

### Test of moderation mediation model

**Fig. 2:**
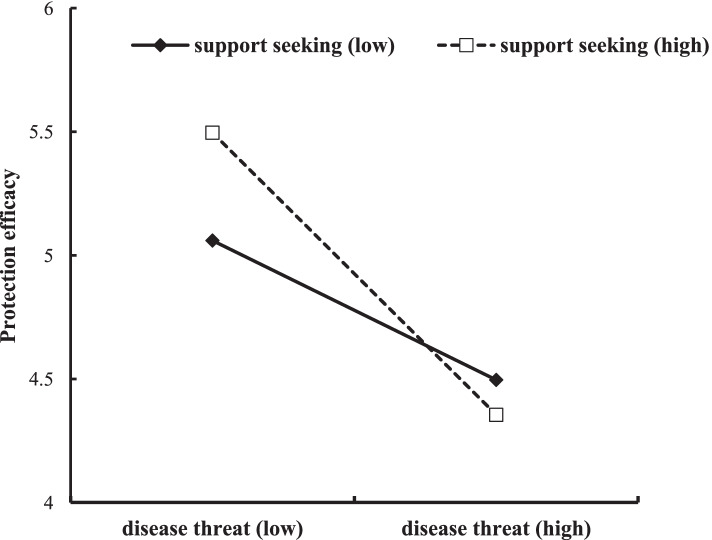
Support seeking as a moderator in the relationship between perceived COVID-19 threat and protection efficacy

The moderated mediation hypothesis was that the indirect effect (mediated pathway) would be moderated by support seeking, after controlling for gender and age. Specially, we tested whether support seeking moderated the indirect effect (with the moderator affecting the first part of the mediated pathway) using the PROCESS macro (Model 7). Table 2 summarizes the results from SPSS macro PROCESS. The results showed that the interaction of perceived COVID-19 threat and support seeking had a significant effect on perceived protection efficacy (*β* = − 0.19, *p* < 0.01). This suggested that the support seeking moderated the indirect effect of perceived COVID-19 risk on xenophobia. We also calculated conditional indirect effects of perceived COVID-19 threat on protection efficacy at different levels of support seeking. The results indicated that the moderated mediation effect was statistically significant at three levels of support seeking (− 1 *SD*, 0, and + 1 *SD*; see Table 3). Simple slope tests revealed that the indirect effect was stronger for individuals with less support seeking (1 SD below the mean) than those with more support seeking (1 *SD* above the mean) (see Fig. 2). Thus, H3 was supported.

**Table 2:** Moderated mediation effect outcomes

	*β*	*SE*	*t*	*P*	LLCI	ULCI
Outcome: Protection efficacy						
Gender	−0.18	0.05	−3.35	< 0.001	−0.28	− 0.07
Age	0.01	0.003	1.77	0.08	−0.01	0.01
Disease threat	−0.62^***^	0.04	−15.38	< 0.001	−0.70	−0.54
Support Seeking	0.25^***^	0.05	4.99	< 0.001	0.15	0.35
Disease threat*Support seeking	−0.19^**^	0.06	−3.13	< 0.01	−0.31	− 0.07
Outcome: Xenophobia						
Gender	0.02	0.07	0.23	0.82	−0.13	0.16
Age	0.01	0.004	1.68	0.09	−0.001	0.02
Disease threat	0.24^***^	0.06	3.90	< 0.001	0.12	0.36
Protection efficacy	−0.09^*^	0.04	−2.12	0.03	−0.17	−0.01

*N* = 1103. Unstandardized regression coefficients were reported

*LLCI* Lower level of the 95% confidence interval, *ULCI* Upper level of the 95% confidence interval

^**^p < 0.01, ^***^p < 0.001

**Table 3:** Significance test of moderated mediation effect

Support Seeking	*β*	Boot SE	Boot CCLI	Boot ULCI
M-SD	0.05	0.02	0.01	0.09
M	0.06	0.03	0.01	0.11
M + SD	0.07	0.03	0.01	0.13

*M* The mean value of support seeking, *SD* The standard deviation of support seeking

## Discussion

The first aim of this study was to examine the relationship between perceived disease threat due to COVID-19 and xenophobia within China toward the people of Wuhan, where the pandemic began. The results showed that those who perceived a greater COVID-19 risk were more likely to show xenophobic attitudes. This finding is in line with previous studies in the West showing that disease threats were associated with greater xenophobia [8–10]. The present study suggested that a xenophobic response could also occur within a large country (e.g., China) when disease threat was salient, as the Chinese public demonstrated significant xenophobic attitudes toward people from Wuhan (a major epidemic area). This provided preliminary evidence that xenophobia could also appear within a country that seems culturally homogeneous under the disease threat.

Consistent with our hypothesis, the results showed that the relationship between perceived COVID-19 risk and xenophobia was due in part to doubts about being able to control the risk of infection. The prior work suggests that protection efficacy mediated the relationship between disease threat and xenophobia response in the context of a low Ebola threat in the U.S [10], our finding, however, indicated that protection efficacy can also serve as an important mediating mechanism explaining how perceived COVID-19 threat translated into xenophobia. This suggest that rational cognitive processes can also happen in a high disease threat (e.g., COVID-19) before an irrational response (e.g., xenophobia) occurs [3]. This finding provided a possible solution to reduce the xenophobia in the current COVID-19 crisis as well as similar situation in the future.

Another finding of the present study was that the indirect effect of perceived COVID-19 threat on xenophobia through lower protection efficacy was moderated by support seeking. Specifically, the first part of the model—the link between disease threat and lower perceived protection efficacy—was weaker among individuals who engaged in more support seeking. Our result supports the buffering effect of support seeking [21], as well as the buffering hypothesis [22], suggesting that more support seeking could provide protection from the negative effects of stressful events. That is, when there is a perception of disease threat, seeking social support can be a buffer against the belief that there is little one can do to reduce the risk of infection. The effect of this interaction in the first link of the mediation process explained part of the overall moderated mediation effect.

There are several possible explanations for this finding. First, support seeking is a coping strategy focused on problem solving [15], and people who seek social support may tend to use a more proactive and practical way to deal with perceived disease threat. This may include asking a doctor about how to prevent infection, asking friends if they know where to buy more masks, and trying to get emotional support from friends or relatives. Support seeking may thus generate rational solutions to cope with the threat, reduce fear about the disease, and decrease biased attitudes towards the out-group. Secondly, support seeking may help individuals to gain more resources (e.g., instrumental and emotional support) [21, 31] than would be available as passive recipients of social support, and thus increase their confidence in dealing with the disease threat. Thirdly, social connection may play an important role in coping with perceived disease threats. For example, in one study individuals who were most psychologically isolated from others showed the strongest xenophobic attitudes during the threat of Ebola, while those who tended to build social connections with others showed less xenophobia [10]. Social connection and interaction could provide individuals a sense of security and belonging in the face of disease threats. Support seeking, to a certain extent, promotes social interaction and connection with other people, and thus strengthens perceived protection efficacy and further reduces the influence of perceived disease threat on xenophobic attitudes.

Our findings make two contributions to the existing literature. First, previous studies mainly focused on xenophobic attitudes in western cultures, indicating that the public showed greater prejudice toward immigrants or foreigners when disease threat (e.g., Ebola and avian influenza) was salient [8, 10]. The results of the present study suggest that the public may also demonstrate xenophobia toward people from areas affected by the epidemic even within the same country. Second, to our knowledge, this was the first study to examine the buffering effect of support seeking in the relationship between disease threat and xenophobia. These findings extend those of previous studies that showed a protective effect of collectivism [10], by confirming the buffering effect of support seeking at the micro–level of the individual.

### Practical implications

The results of this study provide some practical guidance for adjusting to the COVID-19 pandemic. The xenophobic attitudes elicited by perceived disease risk might be reduced by improving people’s perception of protective efficacy and their support seeking behaviors. For example, individuals can be informed (e.g., through internet media and paper media) about the efforts made by authorities and communities to defend against disease threats; people can also be taught what specific actions they can take to protect themselves from infection. These measures may increase individuals’ protection efficacy (e.g., personal and community). In addition, seeking social support may be a practical strategy for dealing with the threat of disease. Policymakers and mental health practitioners may want to encourage the public to seek more social support during this pandemic. Given the nature of infectious diseases, people can achieve this by engaging in indirect contact through internet use or phone communications and so on. The authorities may also provide the public with more opportunities to seek out and receive the social support they need (e.g., online help desks, hotlines). Our findings suggest the value of developing intervention strategies to increase coping with the COVID-19 threat [32]. Furthermore, support seeking enhanced perceived protection efficacy, suggesting that it should be encouraged as a way to reduce other psychological symptoms (e.g., anxiety, stress) [33–35].

### Limitations

There are several limitations to this study. First, the study used a cross-sectional design that precludes the causal inferences. Future researchers may carry out experimental studies to confirm these findings. Second, xenophobia is often viewed as immoral [36], and self-reported measures may be affected by biases like social desirability. Using more comprehensive and objective indicators of xenophobia is recommended in future research. Third, if a participant had not been in the actual presence of someone from Wuhan since the beginning of the pandemic, we asked them to imagine themselves in that situation. This probably biased our results because people might imagine different hazards than those that they would actually experience.

## Conclusion

The present study, conducted in China during the early phases of the COVID-19 pandemic, found that perceived disease risk was associated with greater xenophobia toward people from the Wuhan area, where the pandemic began. This association was explained in part by people’s lack of efficacy about being able to protect themselves from infection. However, perceived COVID-19 risk was not necessarily associated with xenophobic attitudes; people who sought social support in response to the disease threat showed higher protection efficacy, and this buffering effect contributed to the overall process of mediation. The results suggest that strengthening individuals’ protection efficacy and encouraging support seeking may help reduce xenophobia during the COVID-19 pandemic.

## Data Availability

The datasets generated during and/or analyzed during the current study are available from the corresponding author on reasonable request.
